# *Gymnema sylvestre* as a Potential Anti-Inflammatory and Anti-Biofilm Agent Against Anaerobic Infections: An In Vitro Study

**DOI:** 10.3390/plants14040497

**Published:** 2025-02-07

**Authors:** Diego Garcia Miranda, Fernanda Malagutti Tomé, Manuela Maria Viana Miguel, Sabrina Ferreira dos Santos Liberato, Maria Cristina Marcucci, Hugo Vigerelli, Flavia Pires Rodrigues, Cristina Pacheco-Soares, Bruno Henrique Godoi, Florence Carrouel, Luciane Dias de Oliveira, Lucas de Paula Ramos

**Affiliations:** 1 Department of Biosciences and Oral Diagnosis, Institute of Science and Technology, São Paulo State University (UNESP), Francisco José Longo 777, São Dimas, São José dos Campos 12245-000, SP, Brazil; diego.garcia-miranda@etu.univ-lyon1.fr (D.G.M.); fmtome@gmail.com (F.M.T.); vianamiguel.manuela@gmail.com (M.M.V.M.); sliberato95@gmail.com (S.F.d.S.L.); cristina.marcucci@unesp.br (M.C.M.); luciane.oliveira@unesp.br (L.D.d.O.); 2 Neonatology and Neonatal Resuscitation Service, Woman-Mother-Child Hospital, Hospices Civils de Lyon, 59 Boulevard Pinel, 69500 Lyon, France; 3 Multimaterials and Interfaces Laboratory (LMI), CNRS UMR 5615, University Claude Bernard Lyon 1, University of Lyon, 6 rue Victor Grignard, 69622 Villeurbanne, France; 4 Laboratory “Health Systemic Process” (P2S), UR4129, Faculty of Medicine Laennec, University Claude Bernard Lyon 1, University of Lyon, 7 rue Guillaume Paradin, 69008 Lyon, France; florence.carrouel@univ-lyon1.fr; 5 Department of Health Sciences, Paulista University, Highway President Dutra km 157, São José dos Campos 12240-420, SP, Brazil; 6 Center of Excellence in New Target Discovery, Butantan Institute, São Paulo 05503-900, SP, Brazil; hugo.barros@butantan.gov.br; 7 Faculty of Medicine and Health, School of Dentistry, Oral Biology Division, University of Leeds, Leeds LS2 9LU, UK; f.piresrodrigues@leeds.ac.uk; 8 Laboratory of Cell Compartment Dynamics, Institute of Research and Development, University of Vale do Paraíba, Av. Shishima Hifumi 2911, São José dos Campos 12244-000, SP, Brazil; cpsoares@univap.br (C.P.-S.); bhenriquegodoi@gmail.com (B.H.G.); 9 School of Dentistry, Federal University of Alfenas—UNIFAL, R. Gabriel Monteiro da Silva, 700—Centro, Alfenas 37130-001, MG, Brazil

**Keywords:** antimicrobial agents, anti-inflammatory agents, biofilms, Gram-negative anaerobic cocci

## Abstract

This study evaluates the antimicrobial activity of the glycolic extract of *G. sylvestre* against anaerobic pathogens, along with its cytotoxicity, genotoxicity, anti-inflammatory activity, antioxidant effects, and phytochemical composition. Phytochemical analysis was conducted using high-performance liquid chromatography and liquid chromatography–mass spectrometry, while the antioxidant effect was assessed through a DPPH assay. Antimicrobial action was tested on planktonic cultures and biofilms of *Porphyromonas gingivalis*, *Porphyromonas endodontalis*, *Parvimonas micra*, and *Fusobacterium nucleatum*. Cytotoxicity was evaluated using mouse macrophages (RAW 264.7), rat fibroblasts (L929), and human keratinocytes (HaCaT). Anti-inflammatory effects were measured by an immunoenzymatic assay (ELISA) on RAW 264.7 cells. Statistical analysis was performed using a one-way ANOVA and Tukey’s test. Phytochemical analysis revealed the presence of phenolic compounds and flavonoids. The extract demonstrated a reduction of over 95% in biofilms of *P. gingivalis*, *P. micra*, and *F. nucleatum* within 5 min of treatment. Cell viability (HaCaT) remained above 80%. Antioxidant activity showed an EC50 of 353.43 µg/mL, achieving a 50% reduction in free radicals. A significant decrease in TNF-α (a pro-inflammatory cytokine) and an increase in IL-10 (an anti-inflammatory cytokine) were observed. In conclusion, the extract of *G. sylvestre* exhibits promising potential as a therapeutic agent for treating anaerobic infections, inflammation, and oxidative stress.

## 1. Introduction

The therapeutic use of plants has been recognized since ancient times, with records dating back to 2600 BC describing a medicinal system in Mesopotamia that included approximately 1000 plant-based medicines [1,2]. According to the World Health Organization, 85% of the global population depends on medicinal plants for healthcare. In this context, *Gymnema sylvestre*, a perennial woody vine native to Asia, Africa, and Australia and belonging to the Apocynaceae family, has been used in traditional, Ayurvedic, and homeopathic medicine for over 2000 years [3,4,5].

*Gymnema sylvestre* has demonstrated diverse therapeutic properties, including anti-ulcerogenic, antiallergic, anti-stress [6,7], antidiabetic [8], antimicrobial [9], hypolipidemic [10], and anti-obesity effects [11,12]. These therapeutic effects are checked in depth in an attempt to elucidate the chemical components present in the herbal medicine; in this way, liquid chromatography techniques could be identifying phytoconstituents. In the study of plant extracts, it enables the detection and quantification of secondary metabolites such as alkaloids, flavonoids, terpenoids, and phenolics, which are responsible for the various pharmacological properties of plants [13,14]. The stems are rich in oleanolic acid, lupeol, saponins, and stigmasterol [15], while flavones, gymnemic acid, gymnemagenin, gymnemasaponins, and sterols are distributed throughout the plant [9].

In the literature, *G. sylvestre* is recognized for its anti-inflammatory potential [7,8,9]. Jangam et al. [7] investigated its effects in rats with acute respiratory distress syndrome (ARDS) and found that treatment with different doses of *G. sylvestre* extract significantly reduced markers of inflammation and oxidative stress in lung tissues. There was a reduction in the infiltration of inflammatory cells, a decrease in the levels of pro-inflammatory cytokines, and a restoration of the levels of endogenous antioxidants. In addition, molecular analyses revealed that extract negatively modulated the NF-κB and MAPK signaling pathways, which are crucial in mediating inflammatory responses.

Toxicity studies have shown that extract is safe when taken in recommended doses. However, high doses can cause adverse effects such as hypoglycemia, weakness, tremors, excessive sweating, and muscular dystrophy [11,12]. Despite the vast therapeutic potential of *G. sylvestre* extracts, their use requires further scientific evaluation, covering aspects such as antimicrobial activity, anti-inflammatory action, and possible cytotoxic and genotoxic effects.

While the antimicrobial activity of *G. sylvestre* has been studied against clinically significant bacteria, such as *Pseudomonas aeruginosa, Klebsiella pneumoniae, Escherichia coli,* and methicillin-resistant *Staphylococcus aureus* (MRSA) [6,16], its effects on anaerobic bacteria in both planktonic and biofilm forms remain unexplored. Anaerobic bacteria are associated with serious infections and frequently exhibit resistance to standard antimicrobial treatments. According to the National Institutes of Health (NIH), biofilms contribute to 65% of microbial infections and 80% of chronic infections [17,18]. Infectious diseases are among the leading causes of mortality worldwide. Therefore, evaluating the effects of the glycolic extract of *G. sylvestre* on anaerobic bacteria, as well as its cytotoxic, anti-inflammatory, and phytochemical properties, is critical for understanding its potential biological activity.

## 2. Results

### 2.1. Phytochemical Analysis by Gymnema sylvestre Extract

The analysis of total solids in the extract revealed an average concentration of 8.85% with a standard deviation of 0.21, indicating that the extract has a concentration of 88.5 mg/mL. Of these solid compounds, 66.64 ± 0.48 mg/mL was identified as phenolic compounds and 22.94 ± 0.66 mg/mL as flavonoids.

**Figure 1:**
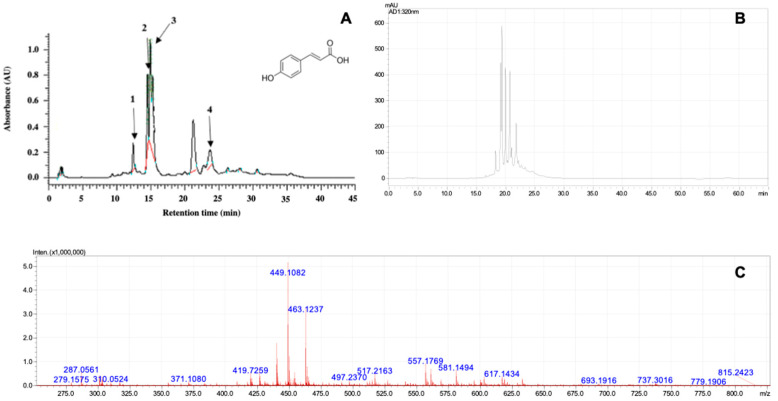
Chromatogram of the glycolic extract of *Gymnema sylvestre* obtained by HPLC (**A**); chromatogram of the glycolic extract of *G. sylvestre* analyzed via LC-MS (**B**); and mass spectrum of the glycolic extract analyzed (**C**).

Figure 1A displays derivatives of p-coumaric acid with retention times (tR) of 14.48 (1); 14.90 (2); 15.24 (3); and 23.67 (4) minutes. The chemical structure of p-coumaric acid is provided “www.sial.com (accessed on 14 May 2024)”. Additionally, quercetin-3-glucoside (*m*/*z* 463) and cyanidin 3-O-glucoside (*m*/*z* 449) were identified, as shown in Figure 1C.

The antioxidant activity analysis demonstrated that 353.43 μg/mL of the extract was required to achieve a 50% reduction in free radicals (EC50).

### 2.2. Determination of Minimum Inhibitory Concentration (MIC) and Minimum Bactericidal Concentration (MBC) for Anaerobic Microorganisms

MIC values could not be determined due to the turbidity of the culture medium used. The MBC results are presented in Table 1.

#### Antimicrobial Action on Monotypic Biofilms

**Figure 2:**
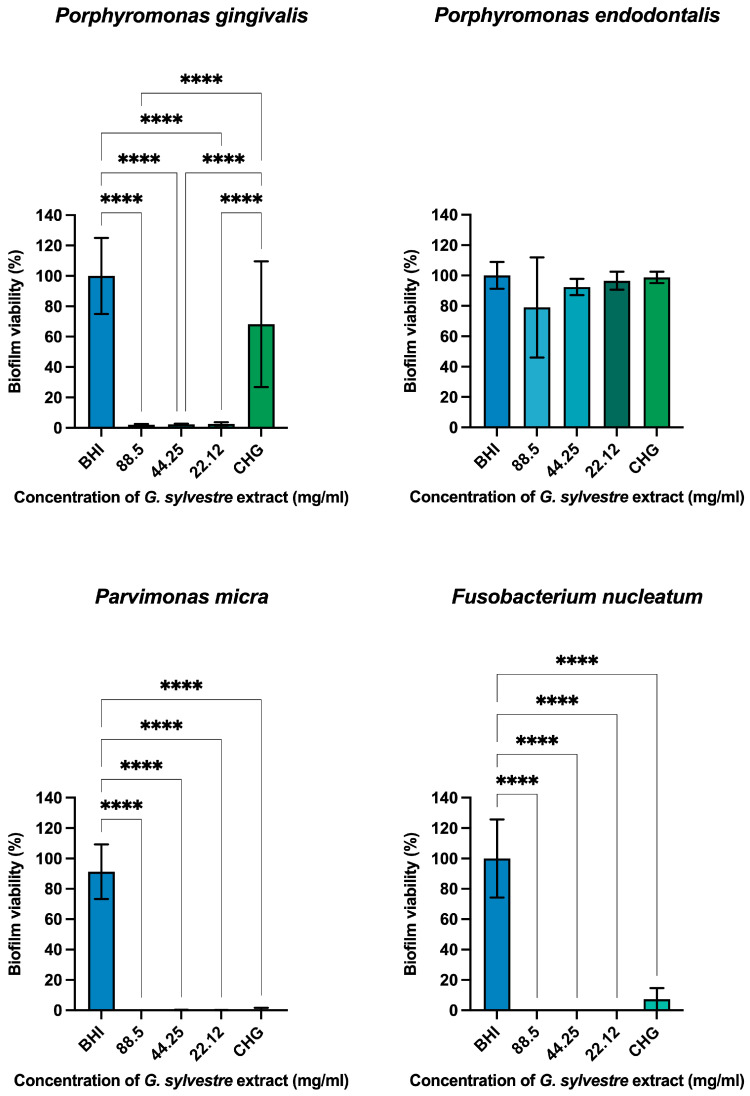
Graphs depicting reductions in monotypic biofilms formed by anaerobic strains after a 5 min treatment with *Gymnema sylvestre* extract, brain heart infusion (BHI), and 0.12% chlorhexidine digluconate (CHG), *p* < 0.0001 (****).

After a 5 min treatment with the extract of *Gymnema sylvestre*, *Porphyromonas gingivalis* exhibited a reduction of over 97.8% at all extract concentrations compared to 31.85% for CHG. *P. endodontalis* showed reductions of 20.99%, 7.06%, and 3.5% for extract concentrations of 88.5, 44.25, and 22.12 mg/mL, respectively, and 1.3% for CHG. *Parvimonas micra* and *Fusobacterium nucleatum* demonstrated reductions exceeding 99% at all extract concentrations compared to 92% for CHG (Figure 2).

**Figure 3:**
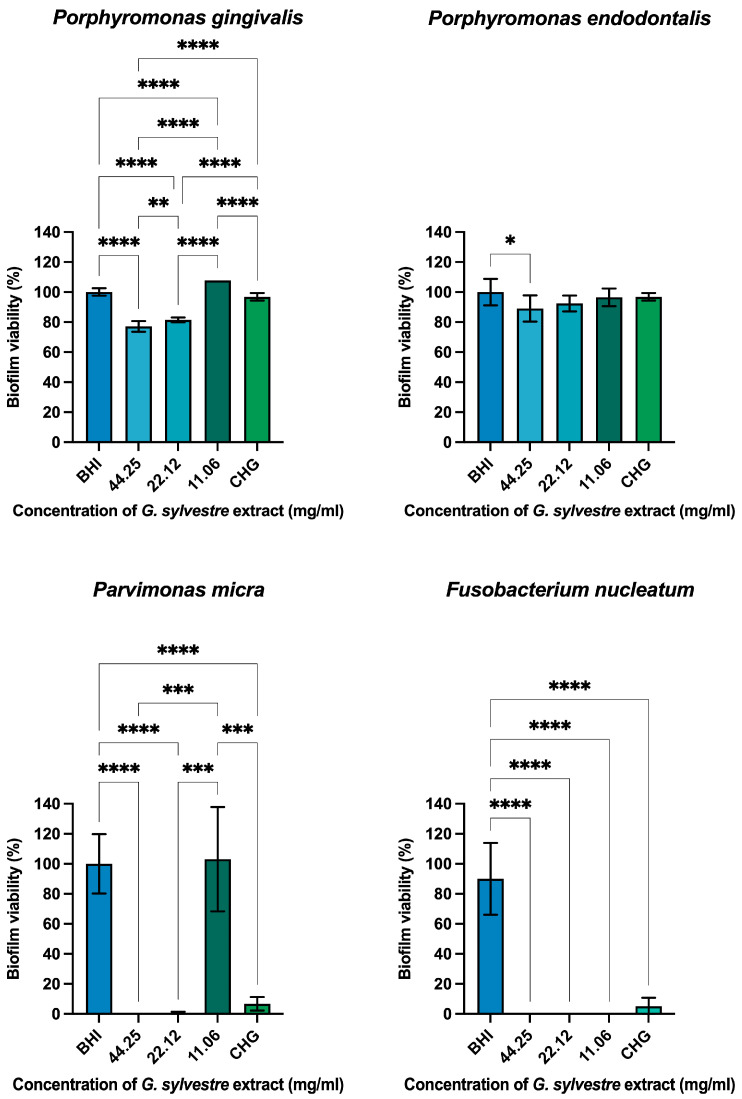
Graphs illustrating reductions in monotypic biofilms by anaerobic strains after 24 h treatment with *Gymnema sylvestre* extract. Brain heart infusion (BHI); 0.06% chlorhexidine digluconate (CHG). Statistical significance is indicated as *p* < 0.0332 (*), *p* < 0.0021 (**), *p* < 0.0002 (***), and *p* < 0.0001 (****).

After a 24 h treatment with the extract of *G. sylvestre*, *P. gingivalis* exhibited reductions of 22.9% and 18.6% for concentrations of 44.25 and 22.12 mg/mL, respectively, and 3.2% for CHG. *P. endodontalis* showed reductions of 10.9%, 7.6%, and 3.6% at concentrations of 44.25, 22.12, and 11.06 mg/mL, respectively, and 3.2% for CHG. *P. micra* demonstrated reductions of 99.9%, 99.3%, and −3.07% at concentrations of 44.25, 22.12, and 11.06 mg/mL of the extract, respectively, compared to 93.2% for CHG. *F. nucleatum* exhibited reductions of more than 99% for all extract concentrations compared to 94.8% for CHG (Figure 3).

### 2.3. Cytotoxicity by MTT Assay

**Figure 4:**
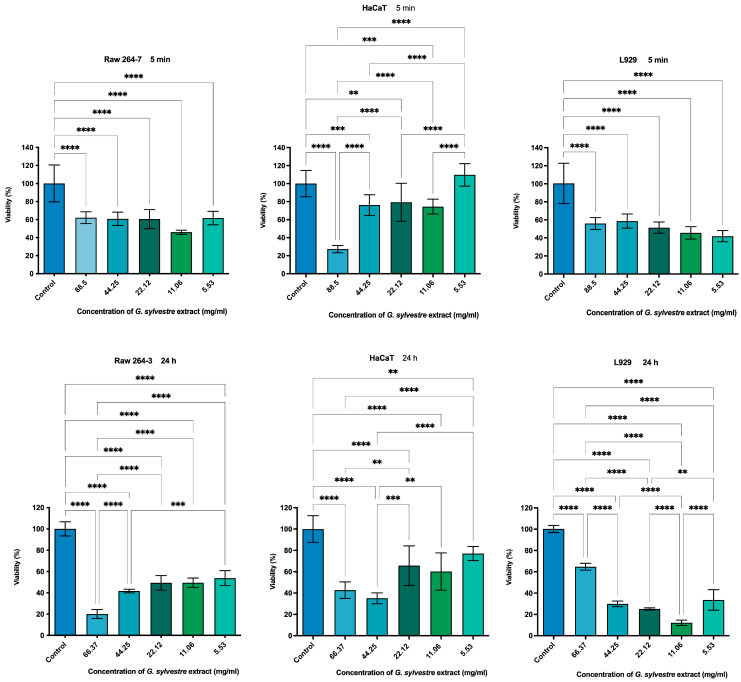
Cytotoxicity induced in RAW 264.7, L929, and HaCaT cells by *Gymnema sylvestre* with 5 min and 24 h of treatment. DMEM + 10% FBS served as the control. Statistical significance is indicated as *p* < 0.0021 (**), *p* < 0.0002 (***), and *p* < 0.0001 (****).

The cytotoxicity of *Gymnema sylvestre* extract was evaluated using the MTT assay on RAW 264.7, HaCaT, and L929 cells incubated with increasing concentrations of the extract for 5 min and 24 h (Figure 4). For RAW 264.7 cells, viability was 62.1%, 60.9%, 60.5%, 45.9%, and 61.7% at 5 min and 20.1%, 41.6%, 49.3%, 49.5%, and 53.7% at 24 h. For HaCaT cells, viability was 27.3%, 76.1%, 79.2%, 74.4%, and 109.6% at 5 min and 42.6%, 35.0%, 65.6%, 60.2%, and 77.0% at 24 h. For L929 cells, viability was 55.9%, 58.6%, 51.4%, 46.6%, and 41.9% at 5 min and 64.7%, 29.8%, 25.1%, 12.1%, and 33.5% at 24 h.

### 2.4. Anti-Inflammatory Analysis by ELISA

**Figure 5:**
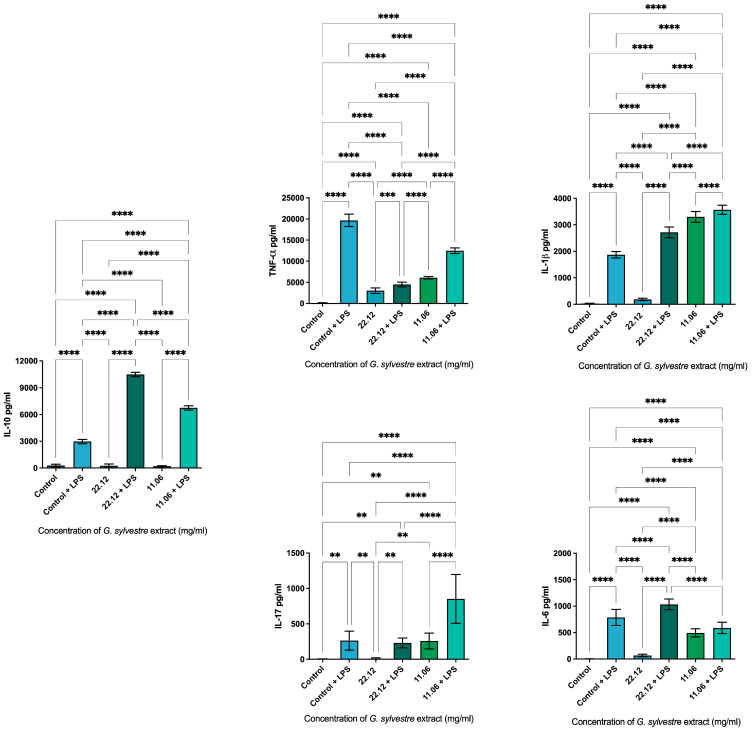
Quantification of pro- and anti-inflammatory cytokines produced by RAW 264.7 cells after 24 h of exposure to the extract. Experimental conditions included DMEM + 10% FBS (control), DMEM + 10% FBS + LPS (control + LPS), Lipopolysaccharide from Escherichia coli (LPS), and *Gymnema sylvestre* extract (GIM). Statistical significance is indicated as *p* < 0.0021 (**), *p* < 0.0002 (***), and *p* < 0.0001 (****).

The anti-inflammatory properties of *Gymnema sylvestre* on RAW 264.7 cells were evaluated by measuring the production of IL-1β, TNF-α, IL-6, IL-17, and IL-10 over a 24 h period, as showed in Figure 5. At an extract concentration of 11.06 mg/mL, IL-1β production increased compared to the positive control, while TNF-α, IL-6, IL-17, and IL-10 levels were lower than the positive control. Upon stimulation with LPS, the levels of IL-1β, IL-17, and IL-10 exceeded those of the positive control. Although the production of IL-6 and TNF-α also increased with LPS stimulation, these levels remained below those of the positive control.

At an extract concentration of 22.12 mg/mL, the production of IL-1β, TNF-α, IL-6, IL-17, and IL-10 was compared to the positive control. However, in combination with LPS, the production of IL-1β, IL-6, and IL-17 exceeded that of the positive control. LPS stimulation combined with 22.12 mg/mL of extract resulted in lower TNF-α production than the positive control, while IL-10 levels were nearly four times higher than those of the positive control.

## 3. Discussion

This study is the first to evaluate the antimicrobial activity of *Gymnema sylvestre* extract against planktonic culture and biofilms of anaerobic bacteria, which are known for their growth challenges and resistance to standard antimicrobials [19]. Previous studies have demonstrated the antimicrobial potential of *G. sylvestre* extract against other bacteria including *Staphylococcus aureus*, *Bacillus cereus*, *Escherichia coli*, *Pseudomonas aeruginosa*, *Salmonella enterica*, *Haemophilus paragallinarum*, and *Clostridium perfringens* type-A [17,20].

Phytochemical analysis of the *G. sylvestre* extract via HPLC identified derivatives of p-coumaric acid with similar structures but varying retention times. These were identified as gymnepregoside H, gymnepregoside G, gymnepregoside I, and a mixture of 12-O-cinnamoyl-20-O-benzoyl-heptahydroxy-(20S)-pregn-6-enyl-3-O-β-cymaropyranoside-(1-4)-β-cymaropyranoside and 12-O-cinnamoyl-20-O-(E)-2-methyl-2-butenoyl-heptahydroxy-(20S)-pregn-6-enyl-3-O-β-cymaropyranoside-(1-4)-β-cymaropyranoside [21].

The extract demonstrated bactericidal effects on planktonic cultures of *Parvimonas micra, Porphyromonas endodontalis*, and *P. gingivalis.* These findings align with those of Karygianni et al. [22], who reported antimicrobial effects of *Olea europaea* and *Pistacia lentiscus* compounds on odontopathogenic bacteria, including *P. gingivalis*, *P. micra*, and *P. intermedia*. Similarly, Shekar et al. [23] found that ethanolic extracts of *Acacia nilotica*, *Murraya koenigii*, *Eucalyptus hybrid*, and *Psidium guajava* inhibited *Fusobacterium nucleatum* and *P. gingivalis*. Kohli et al. [24] observed comparable reductions in *P. gingivalis* after exposure to *Cocos nucifera* and 2% chlorhexidine.

In biofilm models, *G. sylvestre* extract exhibited significant antimicrobial effects against *P. endodontalis*, *P. gingivalis*, *P. micra*, and *F. nucleatum* at various concentrations. After a 5 min exposure, over 95% reductions in *P. gingivalis*, *P. micra*, and *F. nucleatum* biofilms were observed. Collins et al. [25] demonstrated the antimicrobial action of different commercial mouthwashes in planktonic cultures of *P. gingivalis* and *F. nucleatum* after 1, 3, and 5 min of contact. The selected mouthwashes showed greater efficiency against *P. gingivalis*. Bacterial growth was only observed in the treatment with 100 µL of 0.12% chlorhexidine (Septicon^®^). *F. nucleatum* showed some resistance to 0.12% chlorhexidine (Periogard^®^), 0.12% chlorhexidine + 1.00 xylitol (Lacer^®^), and 0.12% chlorhexidine + xylitol (Oddent^®^).

The 24 h treatment revealed a high sensitivity of anaerobic biofilms to the extract, particularly at concentrations of 22.12 and 44.25 mg/mL. Similar findings were reported by Minami et al. [26], who observed significant reductions in *P. gingivalis* biofilm with methanolic extracts of *Lonicera caerulea* var. *emphyllocalyx*. Previous studies have demonstrated the antimicrobial action of various plant extracts on anaerobic bacteria [27,28,29]. However, few studies have assessed the effect of these extracts on biofilms, making the results of this study pioneering and innovative in this context.

The antimicrobial properties of *G. sylvestre* extract are likely linked to the phenolic compound p-coumaric acid found in various plants, including Prunus mume and Ocotea minarum [5,30,31]. This compound has demonstrated efficacy against bacteria and fungi [32,33,34], with its antibacterial activity attributed to altering cell membrane permeability and DNA binding [35]. Molecular docking studies suggest that the inhibition of gyrase may be the mechanism of antibacterial action, while the inhibition of 14α-desmethylase may be responsible for antifungal action; these findings suggest that the caffeic acid derivatives studied are a potential effective antimicrobial agent and could serve as a basis for the development of new drugs [36].

Despite the many applications of *Gymnema sylvestre* extract, such as hypoglycemic activity and cholesterol reduction, there are few studies on its cytotoxicity. Ogawa et al. [37] evaluated the toxicity of *G. sylvestre* leaf extract in male and female Wistar rats for 52 weeks. During this period, no changes were observed in body weight, food consumption, hematology, blood biochemistry, and histopathology, and no mice died. It was concluded that there was no toxic effect in rats treated with the extract in the diet for 2 weeks.

Our results showed a dose-dependent relationship on RAW 264.7 cells, with exposure for 5 min and 24 h. The best viability results were obtained at the lowest concentrations. Similar results were observed by Tahir et al. [38], who evaluated the cytotoxicity of hexane, ethanolic, chloroform, and aqueous extracts of *Gymnema sylvestre* on Vero cells after incubation for 48 h. In their study, the treatments that showed the best results were ethanolic extract at 63.67 µg/mL, chloroform extract at 903.13 µg/mL, hexane extract at 11.33 µg/mL, and aqueous extract at 390.63 µg/mL. These treatments showed cell viabilities of 68.80%, 68%, 65.58%, and 63.95%, respectively. The percentage of viable Vero cells (65.58%) when treated with aqueous extract at 390.63 µg/mL was similar to that obtained in this study by the 24 h contact of the glycolic extract on HaCaT cells (65.6%) at an equivalent concentration (22.12 mg/mL).

Analysis was also performed with epithelial cells (HaCaT), where cell viability was greater than 75% at concentrations of 44.25, 22.12, 11.06, and 5.53 mg/mL in 5 min, and 5.53 mg/mL in 24 h. The extract showed toxicity to fibroblasts (L929) at all evaluated concentrations. When comparing the cytotoxic effects of keratinocytes and fibroblasts, it is possible to see the greater sensitivity of the connective tissue cell. One of the factors related to this is the greater resistance of keratinocytes to chemical or physical aggressions (solar radiation). Keratinocytes have biochemical and molecular apparatuses that interact with the oxidative stress produced by chemical substances [39]. There are reports in the literature of the cytotoxicity of ethanolic extract of *G. sylvestre* on HepG2, K562.HepG2, and A375 cells [40,41].

In addition to cytotoxicity, our study evaluated the genotoxic action of the extract on the same cell lines. Our results suggested that the extract was genotoxic. Similar data were reported by Vannini et al. [41], who evaluated the genotoxicity of three ethanolic extracts of *Gymnema sylvestre* on two cell lines (HepG2 and K562.HepG2).

The anti-inflammatory activity of *Gymnema sylvestre* extract at different concentrations was evaluated on RAW 264.7 cultures exposed to LPS (1 µg/mL) to stimulate the production of pro-inflammatory cytokines (IL-1β, TNF-α, IL-6, IL-17) and anti-inflammatory cytokines (IL-10). Previous studies have evaluated the anti-inflammatory action of the extract [42,43].

Aleisa et al. [44] experimentally induced ulcerative colitis in Wistar rats and pre-treated them with three different doses of the extract (50, 100, 200 mg/kg per day) for 7 days. The production of IL-1β at all concentrations evaluated was statistically lower (*p* < 0.01) compared to the untreated group, while the production of TNF-α and IL-6 was lower when compared to the untreated group only at concentrations of 100 and 200 mg/kg of the extract.

In the study by Jangan et al. [7] investigating the therapeutic effect of *Gymnema sylvestre* hydroalcoholic extract against LPS-induced lung injury, it was found that IL-6 levels were significantly increased in samples from the LPS control group, while treatment with hydroalcoholic extract significantly reduced IL-6 levels. The ELISA results also revealed that treatment significantly reduced IL-1β and CCL2 levels. It was also found that the protein expression data validated the gene expression results and confirmed the protective action of the hydroalcoholic extract of *G. sylvestre* against LPS-induced cytokine expressions, corroborating the data from the present study.

The results obtained in our study showed that at concentrations of 11.06 and 22.12 mg/mL of the glycolic extract of *G. sylvestre*, the production of IL-1β was greater than that of the control group. However, the levels of TNF-α were lower than those of the control group at all concentrations evaluated, and only the lowest concentration of the extract (11.06 mg/mL) reduced the production of IL-6. The ethanolic extract of *G. sylvestre* reduced the levels of TNF-α, IL-1β, and IL-6 in rats with diabetic neuropathy. The best results were obtained with a 100 mg/kg treatment, but the lowest dose evaluated (50 mg/kg) resulted in significant inhibition of IL-6 levels [45].

In addition to decreasing the production of pro-inflammatory cytokines, our study revealed that the glycolic extract of *G. sylvestre* increased the levels of IL-10 at all concentrations evaluated, obtaining the best result at the concentration of 22.12 mg/mL, demonstrating the anti-inflammatory potential of the extract. Moreover, it could be interesting to test *G. sylvestre* extract in combination with other preventive treatments such as ozone [45] and photobiomodulation [46] to understand their mutual effect on biofilm management.

Khan et al. [13] attributed the anti-inflammatory action of *G. sylvestre* to the presence of anthraquinones, Lupeol, and flavonol glycoside. Previous studies support the anti-inflammatory potential of these molecules [47,48,49,50,51].

Future studies should focus on isolating phytoconstituents to elucidate the precise mechanism underlying the antimicrobial activity, to determine which cell structure or gene is affected by the extract, and to identify aspects related to the immunobiochemical mechanism involved in immunomodulation.

## 4. Materials and Methods

### 4.1. Chemical Reagents

The glycolic extract of *G. sylvestre* (Lot: ALL056124, All Chemistry Company do Brazil LTDA^®^, São Paulo, Brazil); formic acid (CAS nº: 64-18-6, purity: 98%, Sigma-Aldrich^®^, St. Louis, MO, USA); methanol (CAS nº: 67-56-1, purity: 99.8% Synth^®^, Diadema, Brazil); ethanol (CAS nº: 64-17-5, purity:99.5%, Synth^®^, Diadema, Brazil); Folin–Ciocalteau reagent (Sigma-Aldrich^®^, St. Louis, MO, USA); sodium carbonate (CAS nº: 497-19-8, purity: 99%, Sigma-Aldrich^®^, St. Louis, MO, USA); aluminum chloride (CAS nº: 7446-70-0, purity: 98%, Sigma-Aldrich^®^, St. Louis, MO, USA); diphenyl picrylhydrazyl radical (DPPH) (CAS nº: 1898-66-4, purity:100%, Sigma-Aldrich^®^, St. Louis, MO, USA); Brucella (BBE) Agar and Broth (BD-Heidelberg, Germany); hemin (CAS nº: 16009-13-5, purity: 96%, Sigma-Aldrich^®^, St. Louis, MO, USA); vitamin K (CAS nº: 58-27-5, purity: 99.8%, Sigma-Aldrich^®^, St. Louis, MO, USA); a sterile physiological solution—0.9% NaCl—(LGC Biotechnology^®^, Cotia, Brazil); Eagle’s medium modified by Dulbecco (DMEM) (LGC Biotechnology^®^, Cotia, Brazil); fetal bovine serum (FBS) (Invitrogen^®^, New York, NY, USA); phosphate-buffered saline (PBS) (Sigma-Aldrich^®^, St. Louis, MO, USA); 3-(4,5-Dimethyl-2-thiazolyl)-2,5-diphenyl-2H-tetrazolium bromide powder (MTT) (CAS nº:298-93-1, purity: 97.5%, Sigma-Aldrich^®^, St. Louis, MO, USA); dimethyl sulfoxide (DMSO) (CAS nº: 67-68-5, purity:99.9%, Sigma-Aldrich^®^, St. Louis, MO, USA); formaldehyde (CAS nº: 50-00-0, concentration: 37%, Synth^®^, Brazil); lipopolysaccharides from *Escherichia coli* O111:B4 (LPSs) (Sigma-Aldrich^®^, St. Louis, MO, USA); ELISA kit Duo set (R&D Systems, Miniápolis, MN, USA); Tween 20 (CAS nº 9005-64-5, Sigma-Aldrich^®^, St. Louis, MO, USA); bovine serum albumin (BSA) (CAS nº 9048-46-8, Sigma-Aldrich^®^, St. Louis, MO, USA); and sulfuric acid (CAS nº 7664-93-9, purity: 97%, Sigma-Aldrich^®^, St. Louis, MO, USA) were used.

### 4.2. Equipment

High-performance liquid chromatography (HPLC) with a photodiode detector instrument HPLC–DAD D-7000 (Merck-Hitachi D-7000, Tokyo, Japan); Electrospray-Ion Trap–Time of Flight ESI-IT-TOF (Shimadzu Co., Quioto, Japan) equipped with a binary Ultra-Fast Liquid Chromatography system UFLC, 20A Prominence (Shimadzu Co., Quioto, Japan); a spectrophotometer (Lonza Biotek^®^, ELX808LBS, Winooski, VT, USA); stirrer (Miulab^®^, Micro plate shaker MIX-1500, Hangzhou, China); water bath precision (Termo Fisher Scientific^®^ TSGP02, Waltham, MA, USA); anaerobic chamber (Whitley DG250 Workstation, United Kingdom; Ultrasonic homogenizer (Sonoplus HD 2200, Bandelin Electronic, Berlin, Brandenburg, Germany); CO_2_ incubator (Sanyo^®^, MCO-19AIC(UV)^®^, Osaka, Japan); and inverted microscope (Zeiss^®^, Axiovert 40C, Jena, Thuringia, Germany) were used.

### 4.3. Extract and Phytochemical Analysis

The glycolic extract of *G. sylvestre* leaves was commercially obtained from All Chemistry Company do Brazil LTDA^®^ (Lot: ALL056124). The extraction of the compounds was carried out by percolation, with the proportion of 20% of the weight of the vegetable leaf and 80% of the weight of propylglycol. The extract was analyzed for soluble solid concentration, total phenolic compounds, and tannins. HPLC–DAD analysis was also carried out to determine the phytochemical composition.

#### 4.3.1. Soluble Solids Content in Ethanol

Three 25 mL beakers were weighed, and their weights were recorded. Five milliliters of the extract was pipetted into each beaker and dried in an oven at 80 °C. After drying, the beakers were placed in a desiccator to cool and then reweighed. The percentage of soluble solids in the extract was calculated using the following formula: % soluble solids (*m*/*m*) = % soluble solids (*m*/*v*)/density.

#### 4.3.2. Determination of Total Phenol Content

One milliliter of the extract was added to a 100 mL volumetric flask, dissolved in 4 mL of ethanol, and diluted with 95 mL of distilled water under stirring to prepare a stock solution. This procedure was performed in triplicate. In a 10 mL volumetric flask, 5 mL of distilled water, 800 μL of Folin–Ciocalteau reagent, and 200 μL of the stock solution were added. The mixture was stirred, followed by the addition of 1.2 mL of a 20% sodium carbonate–tartrate buffer solution. The flask was filled with water to the meniscus. The solution was kept in a water bath at 20 °C. After 2 h, the final volume was adjusted at 20 °C, shaken, and the absorbance was measured at 760 nm using a spectrophotometer. The phenolic content was calculated as gallic acid equivalents (GAE/g) of dry plant material based on a standard curve of gallic acid (5–500 mg/L, Y = 0.0027x − 0.0055, R^2^ = 0.9999).

#### 4.3.3. Determination of the Total Flavonoid Content Expressed as Quercetin

For the determination of the total flavonoid content in the extracts, a stock solution of 100 μL of the glycolic extract was prepared in a 10 mL volumetric flask where methanol was added until the meniscus was complete (stock solution). The procedure was performed from this point in triplicate. A 200 μL aliquot of this solution was withdrawn and transferred to a 10 mL flask already containing 5 mL of methanol. A total of 200 mL of aluminum chloride (AlCl_3_) was added, and the volume was brought to about 10 mL with methanol. The solution was stirred and placed in the water bath for 30 min at 20 °C. After this time, the meniscus was hit, and the absorbance was read at 425 nm. The concentration of total flavonoids expressed in quercetin (% *w*/*w*) was determined by linear regression, calculated from the calibration plot (Y = 0.0162x + 0.0044, R^2^ = 0.999) and expressed as mg quercetin equivalent (QE)/g.

### 4.4. Evaluation of Antioxidant Activity

Eleven tubes were prepared, labeled 0 to 10. Each tube contained 1 mL of 0.30 mM DPPH ethanolic solution and 1 mL of wild-type extract diluted in ethanol at different concentrations as follows: tube 1: 0.01%; tube 2: 0.005%; tube 3: 0.0025%; tube 4: 0.00125%; tube 5: 0.000625%; tube 6: 0.0003125%; tube 7: 0.00015625%; tube 8: 0.00007812%; tube 9: 0.00003906%; and tube 10: 0.00001953%. The control tube (0) contained only the DPPH solution and was used to calibrate the spectrophotometer. The tubes were shaken for 1 min, and absorbance was measured at 515 nm using a spectrophotometer 30 min after the reaction. A graph of absorbance reduction (%) versus extract concentration (μg/mL) was plotted, and the EC50 (μg/mL) was calculated using the least-squares method in a spreadsheet.

### 4.5. Liquid Chromatographic Analysis

HPLC–DAD was utilized to characterize the profile and quantify the content of markers in the extracts. The analysis was conducted using high-performance liquid chromatography with a photodiode array detector and a D-7000 Merck-Hitachi automatic injector and the C18 column Lichrochart-Lichrospher 100 RP-18 for the separation at 5 μm, 12.5 cm. Chromatographic conditions included a mobile phase consisting of a water–formic acid solution (95:5, solvent A) and chromatographic-grade methanol (Merck, solvent B). The flow rate was set to 1 mL/min with a linear gradient, starting at 0% B and ending at 70% B over a runtime of 50 min. Detection wavelengths were set at 280 nm and 340 nm.

#### Liquid Chromatography Coupled to Mass Spectrometry

An Electrospray-Ion Trap–Time of Flight (ESI-IT-TOF) (Shimadzu Co., Japan) equipped with a binary Ultra-Fast Liquid Chromatography system (UFLC, 20A Prominence, Shimadzu) was employed. Samples were loaded in a C18 column (Discovery C18, 5 μm; 50 × 2.1 mm^2^) in a binary solvent system as follows: (A2) water/acetic acid (999/1, *v*/*v*) and (B2) ACN/water/acetic acid (900/99/1, *v*/*v*/*v*). The column was eluted at a constant flow rate of 0.2 mL.min^−1^ with a 0 to 40% gradient of solvent B2 over 35 min. The eluates were monitored by a Shimadzu SPD-M20A PDA detector before introduction into the mass spectrometer. The interface voltage was adjusted to 4.5 KV and the capillary voltage was 1.8 KV at 200 °C. MS spectra were acquired under positive mode and collected in the 350–1400 *m*/*z* range. MS/MS spectra were collected in the 50–1950 *m*/*z* range.

### 4.6. Antimicrobial Analysis

Antimicrobial tests were also carried out on the American Type Culture Collection (ATCC) anaerobic strains of *Porphyromonas gingivalis* (W83), *Porphyromonas endodontalis* (35406), *Parvimonas micra* (33270), and *Fusobacterium nucleatum* (25586).

#### 4.6.1. Determination of Minimum Inhibitory Concentration (MIC) and Minimum Bactericidal Concentration (MBC) for Anaerobic Microorganisms

Tests on planktonic cultures were performed following the protocol M11-A7 according to the Clinical & Laboratory Standards Institute (CLSI) [52]. For this, microbial solutions were prepared from colonies reactivated on Brucella Agar with 1% hemin and 1% vitamin K for 48 h with incubation in an anaerobic chamber. Microbial suspensions were standardized at 0.5 on the McFarland scale. Afterward, the dilution of the extract was prepared in a 96-well microplate, using Brucella Broth enriched with 1% hemin and 1% vitamin K, using N = 10 for each microorganism, performing the tests always in duplicate. After the addition of standard microbial suspensions, 10 μL of the microorganism suspension per well was added. The final concentrations of the extract ranged from 22.12 to 0.04 mg/mL. Finally, the plate was incubated in an anaerobic chamber for 48 h at 37 °C. The identification of MBC was carried out by sowing 10 μL from each well of the microplate, with a subsequent evaluation of colony growth, determining the MBC at the lowest concentration that did not show colony growth.

#### 4.6.2. Antimicrobial Action on Monotypic Biofilms

Microbial suspensions standardized at 0.5 on the McFarland scale were distributed in 96-well microplates in a volume of 100 μL/well. After the distribution of the inoculum, Brucella Broth was added in the same volume, using N = 8 for each test group. The plate was incubated in an anaerobic chamber for 168 h/37 °C, with the replacement of the culture medium every 48 h. After the formation period, the biofilms were exposed to the extract of *G. sylvestre* at concentrations of 88.5, 44.25, and 22.12 mg/mL for 5 min and at concentrations of 44.25, 22.12, and 11.06 mg/mL for 24 h. As a control, Brucella Broth enriched with hemin and menadione was used for both times. After the treatments, the wells were washed twice with PBS, and the biofilms were dispersed using an ultrasonic homogenizer operating at an output power of 30 W for 30 s. Afterward, aliquots were removed from the microplate and dilutions ranging from 10^−2^ to 10^−8^ were performed with subsequent sowing of enriched Brucella Agar. After 48 h of incubation, the CFU/mL count was performed.

### 4.7. Cell Testing

Mouse macrophages (RAW 264.7) and human keratinocytes (HaCaT) from the Rio de Janeiro Cell Bank (BCRJ) in passages 32 and 17, respectively, were grown in DMEM (Dulbecco’s Modified Eagle Medium) supplemented with 10% FBS. The cells were kept in cell culture flasks, incubated in an oven at 37 °C, atmospheric humidity, and 5% CO_2_. To perform the tests, the cells were disaggregated from the bottle and the exclusion test was applied by Trypan blue with automated counter counting.

#### 4.7.1. Cell Viability Test

After counting, 200 μL of DMEM solution and 10% FBS containing 2 × 10^4^ viable cells (DMEM + SFB 10%) were added to each well of 96-well plates, with 24 h incubation for cell adhesion using N = 8 for each group. Then, 10 concentrations of *G. sylvestre* extract were applied, varying from 88.5 mg/mL to 5.53 mg/mL for 5 min and concentrations from 44.25 mg/mL to 2.76 mg/mL for 24 h. DMEM + SFB 10% was used as the control. After the treatments, the wells were washed with sterile PBS and the plates were sent to the MTT test. The metabolic activity of the culture was verified by the reduction method of 3 (4,5-dimethylthiazol-2-yl) 2,5-diphenyltetrazolium in formazan. For this, 5.0 mg of MTT powder was diluted in 10 mL of PBS, then the solution was distributed in the microplate wells at 100 μL/well followed by incubation for 4 h at 37 °C with 5% CO_2_. Subsequently, the supernatant was discarded, followed by the addition of 100 μL/well of dimethyl sulfoxide; after 10 min of incubation, the plates were subjected to reading the optical densities (ODs), which were later converted into a viability percentage.

#### 4.7.2. Anti-Inflammatory Potential in Mouse Macrophages (RAW 264.7)

To assess the anti-inflammatory effects of the extract, 1000 μL of DMEM + 10% SFB solution containing 5 × 10^5^ viable cells (RAW 264.7) were dispensed in 24-well microtiter plates. The plates were incubated (37 °C, 5% CO_2_) for 24 h to promote cell adhesion. Afterward, the cells were exposed to treatment with concentrations of 22.12 and 11.06 mg/mL of *G. sylvestre* diluted in DMEM + 10% FBS. At the same time, 1 μg/mL of lipopolysaccharide (LPS) from *Escherichia coli* was added to each well incubated for 24 h, respecting N = 12 for each test group. Subsequently, the supernatant was collected and stored under refrigeration (−20 °C) with a subsequent quantification of pro-inflammatory cytokines (IL-1β, TNF-α, IL-6, and IL-17) and anti-inflammatory cytokines (IL- 10) by the ELISA method.

The immunoassay was performed following the parameters reported by the DuoSet kit, to which 96-well plates were sensitized with anti-TNF-α, anti-IL-1β, anti-IL-6, anti-IL-10, or anti-IL-17 from mice kept overnight at room temperature. The next day, the plates were washed with PBS containing Tween 20 (PBS-T) and blocked with bovine serum albumin 0.1% for 1 h. Subsequently, the plates received cell culture supernatants (100 μL/well) and cytokine patterns with known concentrations (standard curve). After two hours, the plates were washed (PBS-T) followed by the addition of the biotin-tagged detection antibodies. After incubation, the reaction was developed with a solution containing chromogenic substrate and hydrogen peroxide and blocked after 20 min with 2M sulfuric acid. The optical densities (OD) were read on the spectrophotometer with a wavelength of 450 nm. From the OD, the cytokine levels in (pg/mL) were determined using GraphPad Prism 5.0 software.

### 4.8. Statistical Analysis

The data obtained was analyzed for normality using the D’Agostino, Shapiro–Wilk, and Kolmogorov–Smirnov tests. Data with normal distribution were analyzed using the one-way ANOVA method complemented by the Tukey test. Data without normal distribution were analyzed using the Kruskal–Wallis test supplemented by Dunn’s. Significance levels were *p* < 0.0332 (*), *p* < 0.0021 (**), *p* < 0.0002 (***), and *p* < 0.0001 (****). Statistical analysis was carried out using GraphPad Prism 9.0 software.

## 5. Conclusions

The glycolic extract of *Gymnema sylvestre* demonstrated significant antimicrobial potential. Phytochemical analysis identified phenolic compounds, including four molecules derived from p-coumaric acid. The extract also exhibited antioxidant activity, no cytotoxicity in human keratinocytes (HaCaT) or mouse macrophages (RAW 264.7), and strong anti-inflammatory effects by reducing TNF-α levels, a pro-inflammatory cytokine, while increasing IL-10 levels, an anti-inflammatory cytokine. These findings suggest that *G. sylvestre* extract holds promise as a therapeutic option for managing anaerobic infections, inflammation, and oxidative stress. Further research is needed to evaluate its in vivo applicability and to perform clinical trials for potential drug development.

## Figures and Tables

**Table 1:** Minimum bactericidal concentration of the glycolic extract of *Gymnema sylvestre*.

Microorganism	MBC
*Porphyromonas gingivalis*	22.12 mg/mL
*Porphyromonas endodontalis*	22.12 mg/mL
*Parvimonas micra*	11.06 mg/mL
*Fusobacterium nucleatum*	Absent

## Data Availability

Data is contained within the article.
